# Facile construction of p–n heterojunction Bi_2_O_3_/BNNS for synergistic tetracycline removal through adsorption–photocatalysis

**DOI:** 10.1039/d6ra02440h

**Published:** 2026-05-15

**Authors:** Zhao Du, Yanan Wu, Mengmeng Yu, Zishuang Cheng, Po Hu, Zhonglu Guo, Guifeng Chen, Chengchun Tang, Yi Fang

**Affiliations:** a Provincial Key Laboratory of Intelligent Lighting, Huanghuai University Zhumadian 463000 China hebutdz@163.com; b Hebei Key Laboratory of Boron Nitride Micro and Nano Materials, Hebei University of Technology Tianjin 300130 China fangyi@hebut.edu.cn; c College of Energy Engineering, Huanghuai University Zhumadian 463000 China; d School of Mathematics and Physics, Jingchu University of Technology Jingmen 448000 China

## Abstract

Herein, porous boron nitride nanosheets (BNNS) exhibiting n-type semiconductor characteristics were synthesized *via* high-temperature pyrolysis. Subsequently, a series of Bi_2_O_3_/BNNS composites with superior photocatalytic activity were constructed using a solvothermal method. Experimental results demonstrate that the optimized composite photocatalyst exhibits significantly enhanced adsorption capacity and photocatalytic activity compared to pristine BNNS and Bi_2_O_3_. The optimal Bi_2_O_3_/BNNS composite achieved degradation efficiencies of 94.65%, 94.23%, and 93.97% for tetracycline (TC), oxytetracycline (OTC), and doxycycline (DC) (each at 50 mg L^−1^), respectively, under simulated solar irradiation. This study highlights that the exceptional adsorption capability of BNNS enables the Bi_2_O_3_/BNNS composite to fulfill the requirements for synergistic adsorption–photocatalysis. Furthermore, BNNS serves as an effective growth substrate, significantly regulating the growth of Bi_2_O_3_ nanowires and suppressing their agglomeration, thereby endowing the composite with a large specific surface area and pore volume. The formation of a p–n heterojunction also effectively suppresses the recombination of photogenerated charge carriers within the catalyst. Finally, this work elucidates the detailed process and underlying mechanism of photocatalytic tetracycline degradation driven by simulated sunlight. In summary, this study innovatively employs the Bi_2_O_3_/BNNS composite as a novel photocatalyst for tetracycline degradation and provides theoretical guidance for the design of advanced photocatalysts.

## Abstract 2

A Bi_2_O_3_/BNNS p–n heterojunction was constructed for tetracycline degradation. Under light irradiation, the built-in electric field drives photogenerated electrons to BNNS and holes to Bi_2_O_3_, generating ·O_2_^−^ and ·OH radicals for TC mineralization.

## Graphical abstract

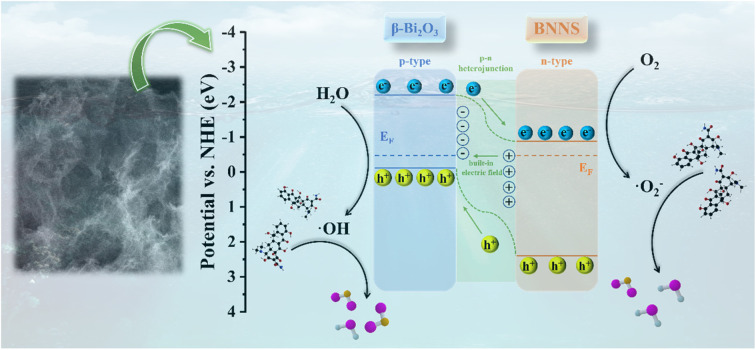

## 1. Introduction

Tetracycline (TC), as one of the most extensively used antibiotics in the world, is widely applied in agriculture and animal husbandry. However, the absorption rate of TC by humans or animals is only 20–30%.^1–3^ A large amount of undecomposed TC will be discharged into the natural environment through various channels, which leads to an increase in the drug resistance of pathogens. This eventually poses a potential risk to the stability of the ecosystem. Photocatalytic technology can utilize sunlight to effectively break down antibiotics into H_2_O, CO_2_, and other small molecules.^4^ However, the low efficiency of photocatalytic in practical applications prevents its large-scale application. Adsorption method is another safe and simple method of antibiotic treatment, while taking into account the advantages of high efficiency.^5,6^ However, this method can only transfer antibiotics from the water to the surface of the adsorbent but cannot completely eliminate the hazards of antibiotics. Therefore, it also has certain limitations in practical application. In order to overcome the above difficulties, the concept of adsorption–photocatalytic synergistic technology has been proposed.^7–9^ The design of multifunctional materials, which possess both efficient adsorption and excellent photocatalytic ability, has become one of the directions to achieving efficient removal for tetracycline pollutants.

Nowadays, metal oxide semiconductors exhibit obvious advantages in the field of photocatalytic degradation. Bismuth-based semiconductor materials are considered to be an ideal class of photocatalysts due to their advantages such as narrower bandgap, less toxicity, and relatively low cost.^10,11^ In recent years, bismuth-based semiconductor materials, like Bi_2_MoO_6_, BiVO_4_, Bi_2_O_3_, Bi_2_O_2_CO_3_, and so on, have been researched more. It is worth mentioning that the hybridization phenomenon between the orbitals of O 2p and Bi 6s^2^ in Bi_2_O_3_ leads to the upward shift of its valence band (VB). Therefore, Bi_2_O_3_ possesses a high oxidation potential and has gained more attention in the field of photocatalytic degradation.^12^ Currently, there are six main crystal morphologies of Bi_2_O_3_: α-Bi_2_O_3_, β-Bi_2_O_3_, γ-Bi_2_O_3_, δ-Bi_2_O_3_, ε-Bi_2_O_3_ and ω-Bi_2_O_3_.^13^ Among them, β-Bi_2_O_3_, with a narrower forbidden bandwidth (∼2.3 eV) and stable structure, has been demonstrated to be a p-type semiconductor catalyst that can be excited by visible light.^14^ However, β-Bi_2_O_3_ also faces the same defect of rapid carrier recombination during photocatalysis. In addition, the β-Bi_2_O_3_, which has a low-dimensional nanostructure, shows better application prospects in photocatalysis due to its higher specific surface area and other factors. However, low-dimensional β-Bi_2_O_3_ also faces the problem of rapid recombination of photogenerated carriers, as well as severe aggregation phenomena, which greatly limits its photocatalytic capability and further development in the field of photocatalysis. In response to these challenges, researchers have proposed various optimization strategies. Among them, constructing the heterostructure can leverage the synergistic effects between different materials to enhance catalytic activity, which is considered one of the ideal modification methods.^15–17^

Hexagonal boron nitride (h-BN) has a stratified constitution akin to graphite, with each layer consisting of numerous hexagonal rings arranged in a regular pattern. Within each hexagonal ring, the atoms of boron (B) and nitrogen (N) alternate their positions, linked by the sublime bond of B–N covalence.^18,19^ These layers are united by the van der Waals. Due to the difference in electronegativity between B and N atoms, the B–N bond, despite being a polar covalent bond, exhibits a certain degree of ionic character.^20^ This endows h-BN with excellent chemical stability, high-temperature stability, and adsorption properties. Currently, some studies have demonstrated that h-BN can effectively inhibit the aggregation of surface nanoparticles, enhance the transfer rate of photo-generated carriers in semiconductor materials, and promote the polarization of tetracycline molecules.^21^ In addition, h-BN, as a non-metallic material, also meets the requirements of photocatalytic technology with its unique environmental friendliness and cost-effectiveness. Porous BN not only possesses the unique physicochemical properties of h-BN, but also its special microstructure shows enormous potential in the field of antibiotic adsorption. Liu *et al.*^22^ successfully synthesized porous BNNS with high specific surface area by calcining the precursor formed by boron trioxide (B_2_O_3_) and guanidine hydrochloride (CH_6_ClN_3_) in hydrogen and nitrogen atmospheres, which demonstrated the excellent adsorption performance of porous BNNS towards tetracycline under different pH conditions. Li *et al.*^23^ used P123 as a structure-directing agent to synthesize a novel porous BN through a two-step method. The porous BN possesses a multimodal micro/mesoporous structure and abundant surface functional groups, allowing for rapid and efficient adsorption of tetracycline molecules in the environment. Song *et al.*^24^ have demonstrated that BN fibers with abundant pore structure possess much higher adsorption capacity for antibiotics compared to commercial BN. Furthermore, it has been substantiated through research that porous BN exhibits the characteristics of an n-type semiconductor.^25^ However, the excessive presence of defect structures in porous BN makes it highly susceptible to the trapping of photogenerated carriers, thereby resulting in an inadequate level of photocatalytic activity. Considering that porous BN can not only suppress the aggregation of low-dimensional Bi_2_O_3_, but also construct a p–n junction with Bi_2_O_3_. Therefore, theoretically, the composite of Bi_2_O_3_ and porous BN can satisfy the requirements of adsorption–photocatalytic synergistic effect.

Therefore, based on the above discussion, and in contrast to previously reported Bi_2_O_3_/BN quantum sheet composites,^26^ this study synthesized two-dimensional porous BN nanosheets (BNNS) with micron-sized lateral dimensions and a wrinkled surface through a high-temperature pyrolysis method. Afterward, one-dimensional β-Bi_2_O_3_ nanowires were grown *in situ* on the surface of BNNS using the solvothermal method, giving rise to a unique “velvet-like” Bi_2_O_3_/BNNS p–n junction heterostructure that has not been previously reported. The experiment results indicate that the Bi_2_O_3_/BNNS composites exhibit excellent tetracycline removal ability under simulated sunlight. This enhanced performance is mainly attributed to the strong adsorption capacity of BNNS for tetracycline and the effective induction of the p–n junction that separates the photo-generated charge carriers in Bi_2_O_3_/BNNS composites. In addition, this work also involves the use of various characterization techniques to conduct in-depth research on the photocatalytic mechanism of Bi_2_O_3_/BNNS composites and the photocatalytic degradation process of tetracycline.

## 2. Experimental section

### 2.1 Materials

Boric acid (H_3_BO_3_, 99.9%, Aladdin Biochemical Technology Co., Ltd), urea (CH_4_N_2_O, 99.9%, Aladdin Biochemical Technology Co., Ltd), bismuth nitrate pentahydrate (Bi(NO_3_)_3_·5H_2_O, 99.9%, Macklin Biochemical Co., Ltd), *N*,*N*-dimethylformamide (C_3_H_7_NO, AR, Tianjin Kemiou Chemical Reagent Co., Ltd), terephthalic acid (C_8_H_6_O_4_, 99%, Macklin Biochemical Co., Ltd), ethanol absolute (C_2_H_6_O, 99.5%, Tianjin Fengchuan chemical Reagent Co., Ltd), tetracycline (TC), doxycycline (DC) and oxytetracycline (OTC) were obtained from BBI Life Science. The chemicals were utilized without undergoing any additional purification processes.

### 2.2 Synthesis of BNNS

BNNS were prepared *via* a two-step method. Firstly, weigh a certain amount of boric acid (H_3_BO_3_) and urea (CH_4_N_2_O) (molar ratio = 1 : 48) and disperse them in a beaker containing 200 mL of pure water. Afterward, heat the mixed solution in a water bath at 70 °C until the water completely evaporates, resulting in the formation of a white powdered precursor. Finally, the precursor is calcined at 1100 °C in nitrogen gas for 4 hours to obtain BNNS powder.

### 2.3 Synthesis of Bi_2_O_3_/BNNS composites and Bi_2_O_3_ nanowire

In a typical synthesis of Bi_2_O_3_/BNNS composites, a certain amount of BNNS was added to 50 mL of C_3_H_7_NO solution and dispersed using ultrasonic waves for 30 min. Then, 0.5 mmol Bi(NO_3_)_3_·5H_2_O and 0.1 mmol C_8_H_6_O_4_ were added into the mentioned solution and stirred for 30 min. Afterward, incubated the mixed solution at 150 °C for 12 h. After cooling to room temperature, the precipitate was washed with C_3_H_7_NO and anhydrous ethanol to obtain the precursor. Finally, Bi_2_O_3_/BNNS composites were synthesized by calcining at 200 °C in the air atmosphere for 4 hours. The composites with Bi_2_O_3_ content of 10, 15, 20, 25 and 30 wt% are named Bi_2_O_3_/BNNS-*x* (*x* = 1, 2, 3, 4, and 5), respectively.

The preparation process of Bi_2_O_3_ nanowires was similar to Bi_2_O_3_/BNNS composites, with the difference that the addition of BNNS was not required.

### 2.4 Catalysts characterization

The X-ray diffraction (XRD) technique using a D8-advance instrument from Bruker, operating at 40 kV with a Cu Kα radiation source, was employed to determine the physical phase composition and crystal structure of the catalyst. Fourier transform infrared spectroscopy (FT-IR) with a VECTOR22 spectrometer, covering a wavenumber range of 4000–400 cm^−1^, was utilized to analyze the chemical bonds and surface functional groups present in the samples. The surface elements of the catalyst were measured by X-ray photoelectron spectroscopy (XPS, ESCALAB 250Xi). The catalyst was analyzed for morphology using scanning electron microscopy (SEM, Quanta 450FEG, FEI) and transmission electron microscopy (TEM, Talos F200S, FEI). The N_2_ adsorption–desorption isotherm of the catalyst was characterized by Autosorb iQ at 77 K in liquid nitrogen. The UV-vis diffuse reflectance spectra (UV-vis DRS) were obtained by utilizing a UV-vis spectrophotometer (U-3900H, 240–800 nm) to investigate the light absorption characteristics and band gap of the catalyst. The photoluminescence spectra and fluorescence lifetimes of the catalysts were obtained with fluorescence spectrophotometer (F-4500) and steady-state transient fluorescence spectrometer (fluorog-3 join-yvon spectrophotometer), respectively. The composition of the tetracycline solution was analyzed by liquid chromatography-mass spectrometry (LC-MS, Compact, Bruker Scientific Instruments).

### 2.5 Photocatalytic activity

The photocatalytic degradation of tetracycline antibiotics was modeled to investigate the photocatalytic activity of the catalysts, in which the antibiotic species included tetracycline hydrochloride (TC), oxytetracycline hydrochloride (OTC), and doxycycline hyclate (DC), all at concentrations of 50 mg L^−1^. Photocatalytic degradation experiments were conducted in a quartz reactor that allows for the circulation of condensed water. The volume of the antibiotic solution and the mass of the catalyst utilized during the photocatalytic degradation experiments were 100 mL and 20 mg, respectively. Before photocatalytic degradation, the antibiotic solution dispersed with the catalyst was first stirred under dark conditions for 60 min to reach the adsorption equilibrium state. The reactor was then placed under a 300 W Xe lamp to keep the light source about 20 cm from the bottom of the reactor and kept the light for 120 min. During the reaction, 5 mL of the suspension was collected and centrifuged every 20 min, after which the UV-vis absorption spectra of the supernatant were measured. Based on the changes in the absorption intensity of the characteristic peaks, the degradation efficiency of the catalyst for antibiotics can be calculated by eqn (2-1):2-1
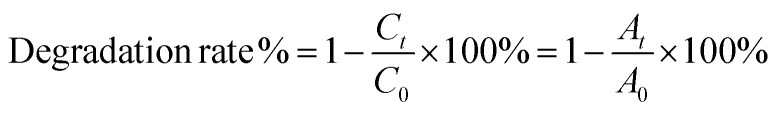
where *C*_*t*_ and *C*_0_ denote the concentration of the antibiotic solution at light times *t* and 0, respectively, and *A*_*t*_ and *A*_0_ are the absorption intensities of the characteristic antibiotic absorption peaks at light times *t* and 0, respectively.

### 2.6 Electrochemical measurements

The photogenerated current curves, electrochemical impedance spectra, and Mott–Schottky plots of various catalysts were evaluated using a three-electrode system on the CEI670 electrochemical workstation. ITO conductive glass, coated with the catalyst, served as the working electrode, while a Pt electrode and an Ag/AgCl electrode functioned as the counter and reference electrodes, respectively. The preparation of the working electrode involved several steps: initially, a specific quantity of catalyst, PVDF, and anhydrous ethanol was thoroughly ground in a mortar. The resultant mixture was then uniformly applied onto the conductive glass substrate. Subsequently, the coated glass was placed in an oven at 60 °C for 12 hours to eliminate excess solvent and ensure the formation of a stable catalyst film. For the electrochemical performance tests, a 0.5 M Na_2_SO_4_ solution was utilized as the electrolyte.

## 3. Results and discussions

**Fig. 1:**
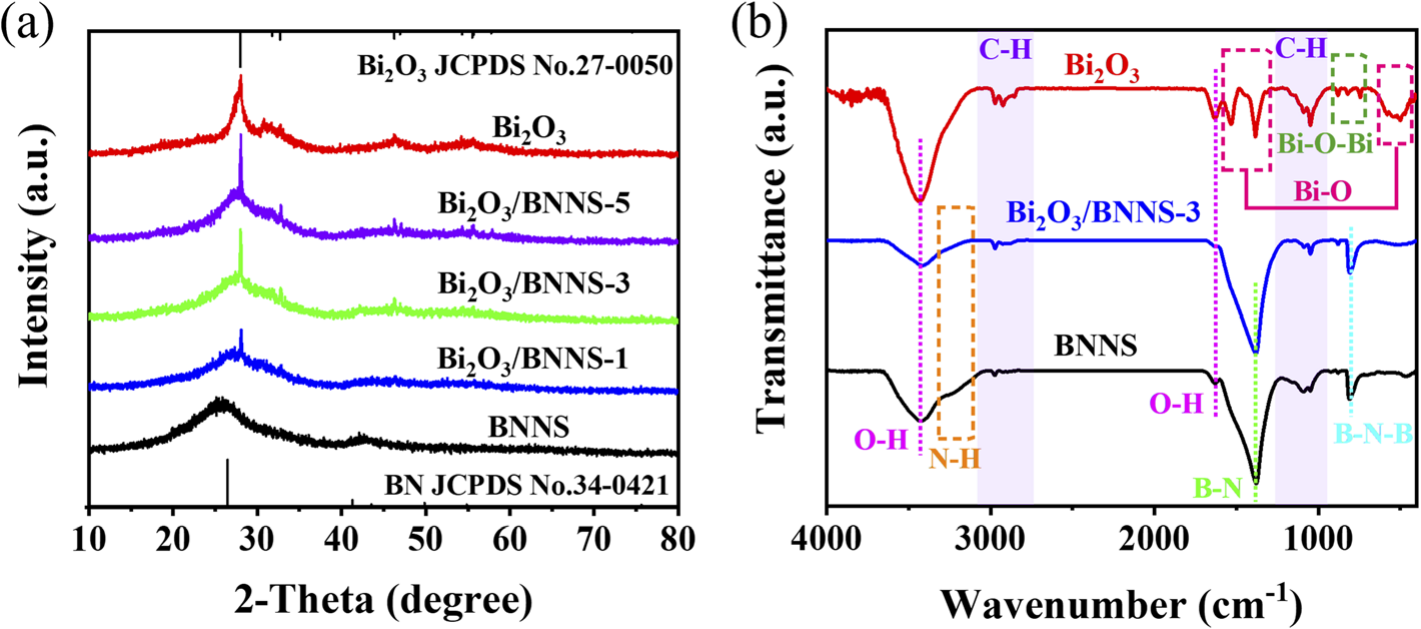
(a) XRD patterns of Bi_2_O_3_, BNNS and Bi_2_O_3_/BNNS composites; (b) FT-IR spectra of Bi_2_O_3_, BNNS and Bi_2_O_3_/BNNS-3.

The crystal structure and composition of the synthesized Bi_2_O_3_ nanowires, BNNS, and composites were analyzed using XRD, as illustrated in Fig. 1a. Bi_2_O_3_ is known to exhibit six distinct crystalline structures. The Bi_2_O_3_ nanowires produced in this study displayed diffraction peaks at 2*θ* = 28.0°, 30.8°, 31.8°, 32.8°, 46.3°, 54.5°, and 55.6°, corresponding to the (201), (211), (002), (222), (400), (203), and (421) planes of β-Bi_2_O_3_ (JCPDS No. 27-0050).^27^ In contrast, for the BNNS, clear diffraction peaks attributed to h-BN (JCPDS No. 34-0421) were identified at the (002) and (100) planes, located at 25.8° and 42.5°, respectively.^28^ In contrast to the ultrathin BN synthesized through the ball milling method, the X-ray diffraction peaks of BNNS exhibit a broader profile, indicating that BNNS has a relatively lower degree of crystallinity and a higher density of defect structures. Additionally, shifts in the diffraction peak positions confirmed the incorporation of dopant elements such as oxygen and carbon within the BNNS structure. In the XRD spectrum of the Bi_2_O_3_/BNNS, both Bi_2_O_3_ and BNNS characteristic diffraction peaks were distinctly observed. The intensity of the Bi_2_O_3_ peaks increased with higher Bi_2_O_3_ content, demonstrating the successful fabrication of the composites. Notably, the full width at half maximum (FWHM) of the Bi_2_O_3_ diffraction peaks in the composite was narrower than pure Bi_2_O_3_, indicating that BNNS acts as an effective growth matrix, enhancing the crystallinity of Bi_2_O_3_. Furthermore, strong interactions between Bi_2_O_3_ and BNNS resulted in slight shifts in the diffraction peaks of BNNS.^29^

The investigation aimed to analyze the functional groups and chemical bonds present in Bi_2_O_3_, BNNS, and Bi_2_O_3_/BNNS through FT-IR analysis. In Fig. 1b, a prominent peak at approximately 3400 cm^−1^ is observed for Bi_2_O_3_, BNNS, and Bi_2_O_3_/BNNS-3, indicating the existence of O–H bonds on the material surfaces. Additionally, absorption bands ranging from 2820 to 3000 cm^−1^ and 950–1200 cm^−1^ are associated with C–H bond vibrations, suggesting a significant presence of –OH on all material surfaces alongside internal carbon elements. Furthermore, an absorption band corresponding to the N–H bond is detected between 3050 and 3260 cm^−1^ for BNNS, indicating the generation of amino groups on the surface during precursor pyrolysis.^30^ These amino groups serve as plentiful hydrogen-bonding ligands, facilitating the formation of a heterojunction between BNNS and Bi_2_O_3_. In the case of the Bi_2_O_3_/BNNS blend, absorption peaks at 425–640 cm^−1^ and 1285–1580 cm^−1^ are related to the Bi–O bonds present within the BiO_6_ octahedra, while the peak at 717–909 cm^−1^ corresponds to Bi–O–Bi stretching vibrations.^31,32^ Moreover, Bi_2_O_3_/BNNS-3 displays distinctive absorption bands typical of Bi_2_O_3_ and additional peaks at approximately 803 and 1380 cm^−1^, representing B–N–B interlayer bending and B–N intralayer stretching vibrations respectively. This observation confirms the successful synthesis of Bi_2_O_3_/BNNS and indicates that the inclusion of BNNS does not impact the chemical structure of Bi_2_O_3_.

**Fig. 2:**
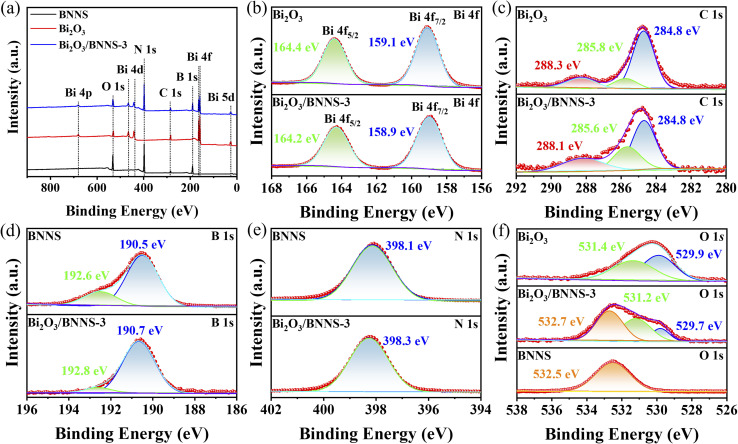
(a) XPS full spectrum, (b) Bi 4f, (c) C 1s, (d) B 1s, (e) N 1s and (f) O 1s high-resolution XPS spectra of Bi_2_O_3_, BNNS and Bi_2_O_3_/BNNS-3.

To perform a comprehensive analysis of the surface elemental composition and chemical states of Bi_2_O_3_, BNNS, and the Bi_2_O_3_/BNNS, XPS spectra of samples were measured and analyzed. The binding energies of the various elements were calibrated using the C 1s peak at 284.8 eV as a reference. The full XPS spectra for Bi_2_O_3_, BNNS, and Bi_2_O_3_/BNNS-3 (Fig. 2a) reveal that the surface of the composites comprises five elements: Bi, O, C, B, and N, with no detectable impurity elements present. Fig. 2b presents the high-resolution XPS spectra for Bi 4f in both Bi_2_O_3_ and Bi_2_O_3_/BNNS-3. The two characteristic peaks observed correspond to Bi 4f_5/2_ and Bi 4f_7/2_, both exhibiting an energy level difference of 5.3 eV for these materials. Notably, the binding energies of Bi 4f_5/2_ and Bi 4f_7/2_ in Bi_2_O_3_ are elevated, recorded at 164.4 eV and 159.1 eV, respectively. In contrast, the binding energies for the composites are slightly lower, measuring 164.2 and 158.9 eV for Bi 4f_5/2_ and Bi 4f_7/2_. The high-resolution XPS spectra of the C 1s for Bi_2_O_3_ and BNNS-3 (Fig. 2c) reveal three characteristic peaks. The peak at 284.8 eV is attributed to the C–C bonds from impurity carbon sources. In Bi_2_O_3_, the peaks at 288.3 eV and 285.8 eV correspond to C–O bonds and Bi–C bonds, indicating the introduction of a small amount of carbon during the preparation process of Bi_2_O_3_.^33^ Similarly, in Bi_2_O_3_/BNNS-3, the binding energies of the C–O and Bi–C bonds (288.1 eV and 285.6 eV) are smaller than those in Bi_2_O_3_. The high-resolution XPS spectra of the B 1s and N 1s for BNNS and Bi_2_O_3_/BNNS-3 (Fig. 2d and e) reveal characteristic peaks of BNNS at 192.6 eV, 190.5 eV, and 398.1 eV, corresponding to B–O, B–N, and N–B bonds, respectively. However, the binding energies of the B–O, B–N, and N–B bonds in the composites (192.8 eV, 190.7 eV, and 398.3 eV) surpass those in BNNS. A comparison of the high-resolution O 1s XPS spectra of Bi_2_O_3_, BNNS, and Bi_2_O_3_/BNNS-3 (Fig. 2f) reveals that the two fitted peaks in Bi_2_O_3_ correspond to Bi–O bonds (529.9 eV) and surface chemically adsorbed oxygen (531.4 eV). Meanwhile, a characteristic peak for the B–O bond is observed at 532.5 eV in BNNS. Conversely, the binding energy of the B–O bond (532.7 eV) surpasses that present in BNNS. This phenomenon reveals the strong interaction between Bi_2_O_3_ and BNNS, which drives the electron transfer within the system. As electrons transfer from BNNS and accumulate on the surface of Bi_2_O_3_, the charge distribution at the interface is reconfigured, thereby forming a built-in electric field. This series of processes confirms the formation of the heterojunction structure.^34^

**Fig. 3:**
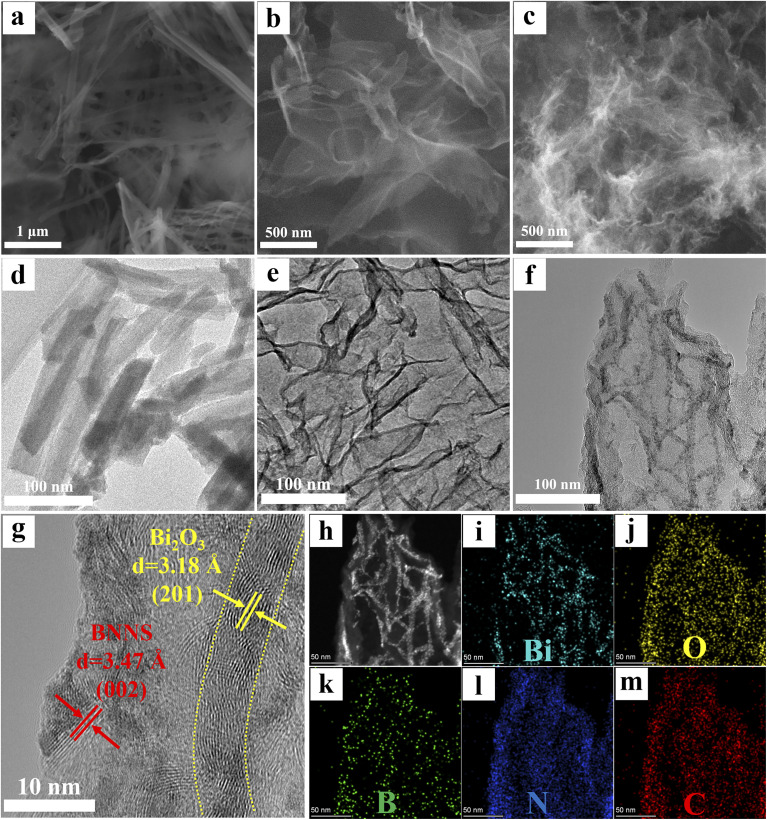
(a) SEM and (d) TEM images of Bi_2_O_3_; (b) SEM and (e) TEM images of BNNS; (c) SEM, (f) TEM, (g) HRTEM, (h) HAADF and (i–m) elements mapping images of Bi_2_O_3_/BNNS composites.

Fig. 3 displays the SEM and TEM micrographs of Bi_2_O_3_, BNNS and Bi_2_O_3_/BNNS. Bi_2_O_3_ exhibits a uniform one-dimensional fibrous structure with a high aspect ratio, alongside noticeable aggregation tendencies. Contrary to SEM findings, the TEM image reveals that the length of Bi_2_O_3_ is approximately 100 nm, while the diameter remains around 30 nm. This could stem from the instability in the structure of Bi_2_O_3_ nanowires, leading to structural disruptions under extended ultrasonication. Fig. 3b and e exhibit the SEM and TEM micrographs of BNNS, depicting an ultra-thin, graphene-like layered configuration with abundant surface creases. Such a morphology grants BNNS a sizable specific surface area, furnishing an ample number of active sites for the nucleation, growth of Bi_2_O_3_, and ensuing photocatalytic transformations. The SEM image of the Bi_2_O_3_/BNNS shows a structure similar to that of BNNS, but upon closer observation, a layer of fine “fuzz” can be seen on its surface. The TEM image (Fig. 3f) showcases a uniform dispersion of nanowires on the surface of BNNS in a more intuitive manner. The HRTEM image (Fig. 3g) intuitively displays the crystal structures of different regions of the composite. Clear lattice fringes are observed in both the sheet-like BNNS and the nanowire structures, with spacings of 3.47 Å and 3.18 Å, corresponding to the (002) plane of h-BN and the (201) plane of β-Bi_2_O_3_, respectively, confirming that the nanowires are composed of Bi_2_O_3_. Notably, the two components are in intimate contact, forming a well-defined heterointerface at the junction between BNNS and Bi_2_O_3_, which confirms the successful construction of the heterojunction. Furthermore, the results indicate that BNNS acts as a growth substrate, effectively regulating the morphology of the Bi_2_O_3_ nanowires and resulting in a significant reduction in both their diameter and length. Fig. 3h–m exhibit the HAADF and element mapping images of Bi_2_O_3_/BNNS, providing further evidence of the homogeneous distribution of Bi_2_O_3_ nanowires on the BNNS surface.

**Fig. 4:**
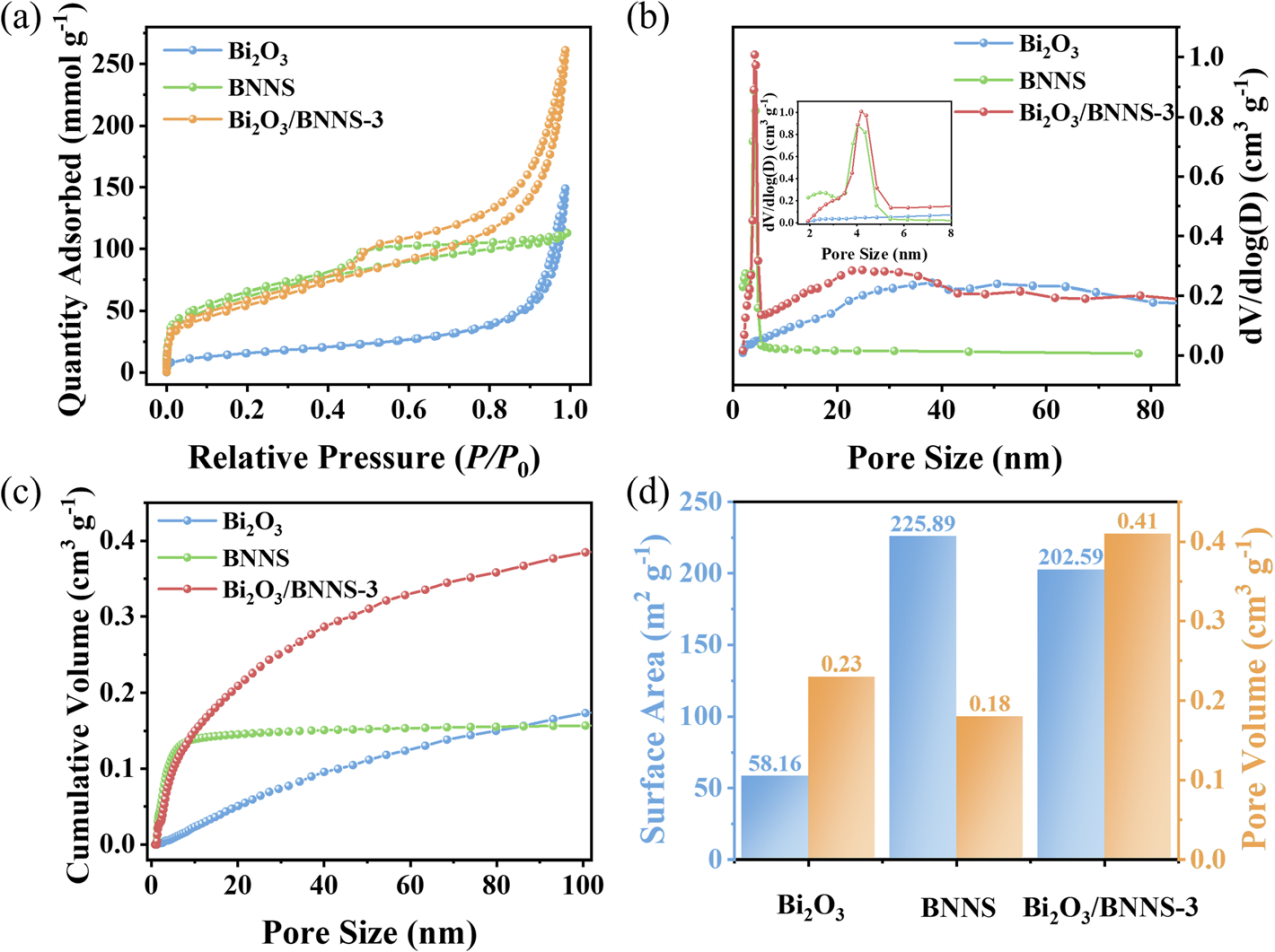
(a) N_2_ adsorption–desorption isotherms, (b) pore size distribution curves, (c) cumulative pore volume curves and (d) specific surface area/cumulative pore volume plots for Bi_2_O_3_, BNNS and Bi_2_O_3_/BNNS-3.

The surface properties of catalysts are one of the main factors affecting the photocatalytic activity. The N_2_ adsorption–desorption isotherms of Bi_2_O_3_, BNNS, and Bi_2_O_3_/BNNS-3 were examined, with results presented in Fig. 4a. Bi_2_O_3_ displayed a Type IV isotherm with an H_3_ hysteresis loop, indicative of its mesoporous nature. Conversely, BNNS demonstrated a Type I isotherm with an H_4_ hysteresis loop, highlighting the presence of abundant micropores and slit-like mesopores resulting from layer-by-layer stacking.^35^ In contrast to the aforementioned materials, the Bi_2_O_3_/BNNS composite material exhibited a typical Type IV isotherm with an H_4_ hysteresis loop, exposing its hierarchical porous structure. Fig. 4b illustrates that Bi_2_O_3_/BNNS-3 displays a narrow pore size distribution centered at 4 nm within the range of 2–8 nm and includes numerous pores with diameters ranging from 8–80 nm. Utilizing the BET method revealed that the cumulative pore volume of Bi_2_O_3_/BNNS-3 (0.404 cm^3^ g^−1^) exceeded that of BNNS (0.175 cm^3^ g^−1^) and Bi_2_O_3_ (0.231 cm^3^ g^−1^) (Fig. 4c and d). Furthermore, the specific surface area of Bi_2_O_3_/BNNS-3 (202.586 m^2^ g^−1^) approximates that of BNNS (225.889 m^2^ g^−1^) but surpasses that of Bi_2_O_3_ (58.162 m^2^ g^−1^) notably (Fig. 4d). The results suggest that the even dispersion of Bi_2_O_3_ nanowires on BNNS surface leads to the formation of additional accumulated pores while preserving the pore features of both BNNS and Bi_2_O_3_. In conclusion, the high pore volume and large specific surface area of composites increase adsorption and reactive sites, enhancing the efficiency of photocatalytic reactions.

**Fig. 5:**
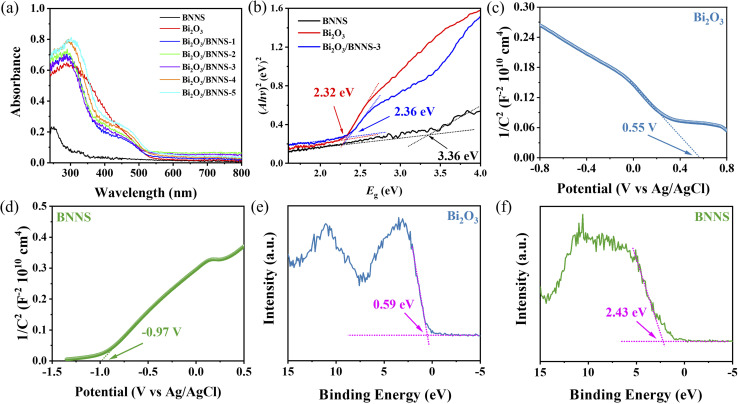
(a) UV-vis absorption spectra of BNNS, Bi_2_O_3_ and Bi_2_O_3_/BNNS composites; (b) bandgap of BNNS, Bi_2_O_3_ and Bi_2_O_3_/BNNS-3; (c and d) Mott–Schottky curves and (e and f) VB-XPS spectra of Bi_2_O_3_ and BNNS.

The UV-visible absorption spectra of BNNS, Bi_2_O_3_, and Bi_2_O_3_/BNNS were analyzed to assess the optical utilization efficiency. As depicted in Fig. 5a, Bi_2_O_3_ demonstrates outstanding visible light absorption capacity with an absorption edge around 550 nm, whereas BNNS exhibits minimal light absorption within the visible spectrum. Nevertheless, the optical absorption properties of both materials significantly improve across the entire spectrum upon their combination. This enhancement can be attributed to the unique microstructure of the Bi_2_O_3_/BNNS, which promotes internal light reflection. Based on the provided test data, the band gaps of Bi_2_O_3_, BNNS and Bi_2_O_3_/BNNS-3 were determined as 2.32 eV, 3.36 eV and 2.36 eV, respectively, through the application of the Kubelka–Munk formula (refer to Fig. 5b). The presence of various additional elements such as C, O and H elements in the raw materials (H_3_BO_3_ and CO(NH_2_)_2_) utilized during the synthesis of BNNS, alongside B and N elements, lead to the incorporation of trace impurities within BNNS. This phenomenon can potentially influence the energy level configuration of h-BN as reported in the literature, consequently resulting in a narrower band gap for BNNS compared to the typical range observed in h-BN (5–6 eV).

Analyzing the band structures of Bi_2_O_3_ and BNNS enhances the understanding of the transfer mechanisms involved with photogenerated carriers in Bi_2_O_3_/BNNS. The Mott–Schottky plots of Bi_2_O_3_ and BNNS (Fig. 5c and d) demonstrate that the negative slope of the Bi_2_O_3_ curve confirms its classification as a p-type semiconductor, consistent with previously reported findings, while the positive slope of the BNNS plot indicates its n-type semiconductor characteristics.^36^ Calculations reveal that the flat band potentials (*E*_fb_) for Bi_2_O_3_ and BNNS are 0.55 V and −0.97 V, respectively, relative to the Ag/AgCl electrode. Given that the flat band potential of a p-type semiconductor approximates its valence band maximum (*E*_VB_), and that of an n-type semiconductor approximates its conduction band minimum (*E*_CB_), and using the previously determined band gaps of Bi_2_O_3_ and BNNS (2.32 eV and 3.36 eV, respectively), the *E*_VB_ and *E*_CB_ of Bi_2_O_3_ are calculated to be 0.75 V and −1.57 V (*vs.* NHE), while the *E*_CB_ and *E*_VB_ of BNNS are −0.77 V and 2.59 V (*vs.* NHE), respectively.

**Fig. 6:**
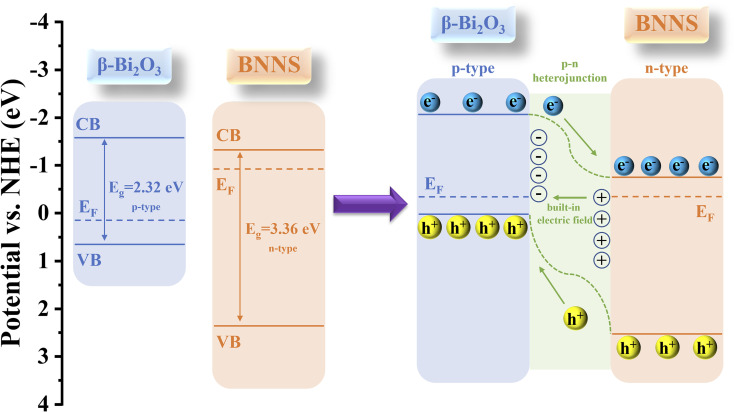
Schematic diagram of the energy band structures of Bi_2_O_3_, BNNS and Bi_2_O_3_/BNNS.

To validate the accuracy of the aforementioned results, the VB-XPB spectra of Bi_2_O_3_ and BNNS were tested and analyzed. The findings are illustrated in Fig. 6e and f, while the calculation formulas for *E*_VB, NHE_ are presented in eqn (2):

1 *E*_VB, NHE_ = *φ* + *E*_VB, XPS_ − 4.44

The power function used in VB-XPS testing, denoted as *φ* (4.60 eV),^37^ facilitates the calculation of the *E*_VB, NHE_ values for Bi_2_O_3_ and BNNS, which are 0.75 eV and 2.59 eV, respectively. Furthermore, the *E*_CB, NHE_ values are −1.57 eV and −0.77 eV, respectively. These results align with those obtained from Mott–Schottky curve calculations, leading to the conclusion that the energy band structures of Bi_2_O_3_ and BNNS are represented in Fig. 6 (left). As a p–n junction forms between Bi_2_O_3_ and BNNS, their Fermi levels (*E*_F_) gradually align, prompting the energy bands of Bi_2_O_3_ and BNNS to shift downward and upward, respectively. As a result (as illustrated in Fig. 6 right), in the dark equilibrium state, the depletion of electrons in n-type BNNS induces a positive space charge at BNNS, while the depletion of holes in p-type Bi_2_O_3_ induces a negative space charge at Bi_2_O_3_, thereby establishing a built-in electric field directed from BNNS to Bi_2_O_3_. Under illumination, driven by this built-in electric field, photogenerated electrons transfer from Bi_2_O_3_ to BNNS and photogenerated holes transfer from BNNS to Bi_2_O_3_, suppressing the recombination of photogenerated carriers.

**Fig. 7:**
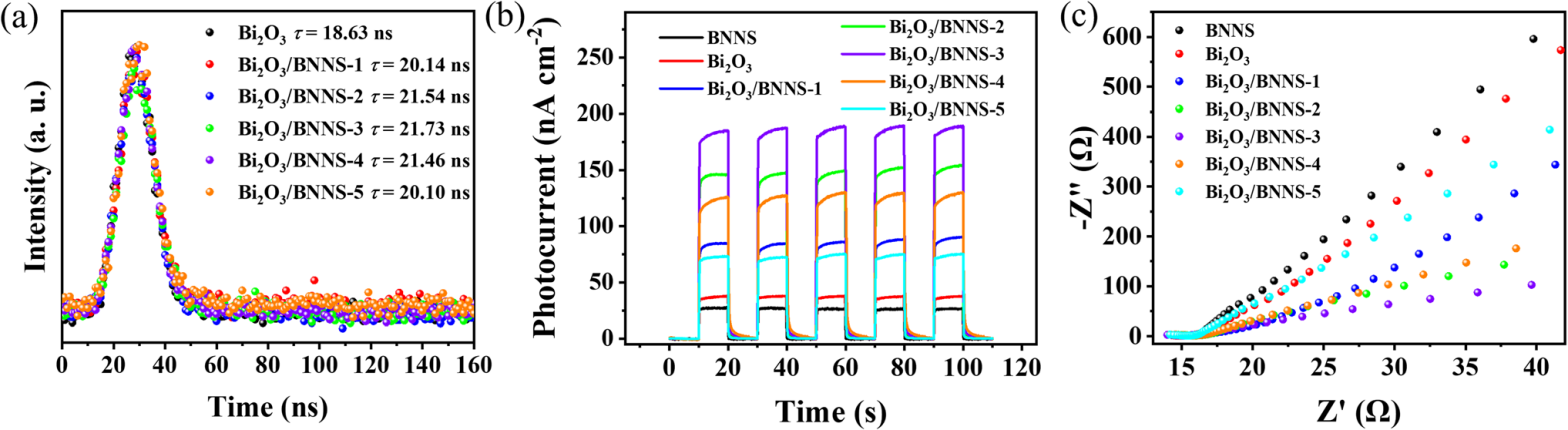
(a) The TRPL spectra of Bi_2_O_3_ and Bi_2_O_3_/BNNS composites; (b) the transient photocurrent curve and (c) electrochemical impedance curves of BNNS, Bi_2_O_3_ and Bi_2_O_3_/BNNS composites.

The separation efficiency of photogenerated charge carriers significantly impacts the photocatalytic performance of catalysts. The recombination kinetics of photo-induced charge carriers in Bi_2_O_3_ and Bi_2_O_3_/BNNS were analyzed using time-resolved photoluminescence spectroscopy (TRPL). As shown in Fig. 7a, fitting the experimental data with a double exponential fitting model the charge carrier lifetimes in the composite materials were all extended compared to Bi_2_O_3_ (18.63 ns), particularly for Bi_2_O_3_/BNNS-3 (21.73 ns). This phenomenon demonstrates that the presence of p–n junctions greatly improves the separation and transport of photogenerated charge carriers in the catalyst. The electrochemical behavior of semiconductor materials can provide insights into the charge carrier separation efficiency. Fig. 7b exhibits the photocurrent responses of BNNS, Bi_2_O_3_, and Bi_2_O_3_/BNNS composites showing that all materials exhibit current generation under illumination, with the composite materials showing higher photocurrent density than BNNS and Bi_2_O_3_. In addition, the electrochemical impedance spectroscopy (EIS) curves of semiconductor materials can be used to study the resistance encountered during the charge transfer process. The results (Fig. 7c) show that the EIS impedance of the composite materials is lower than that of BNNS and Bi_2_O_3_. In conclusion, the photoelectrochemical data further confirm that the existence of internal p–n junctions and built-in electric fields in Bi_2_O_3_/BNNS composites facilitate the establishment of effective charge transfer routes, resulting in superior charge carrier separation rates and efficient charge transfer.

**Fig. 8:**
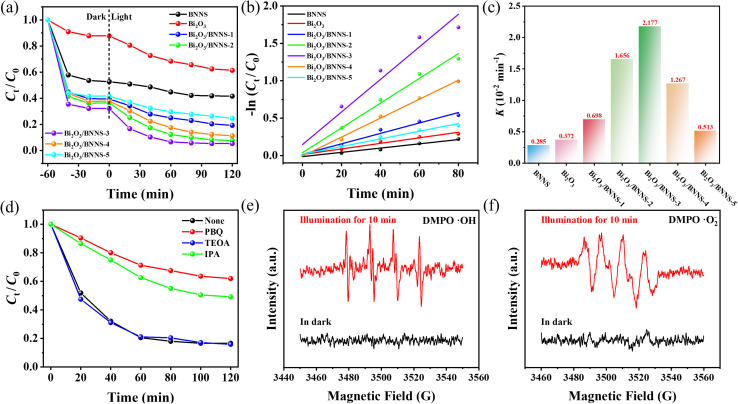
(a) Photocatalytic degradation curves, (b) first level kinetic model fitting and (c) rate constants of BNNS, Bi_2_O_3_ and Bi_2_O_3_/BNNS composites for tetracycline; (d) the photocatalytic degradation of TC curves by Bi_2_O_3_/BNNS-3 after adding different trapping agents, respectively; ESR signals of (e) DMPO-·OH and (f) DMPO-·O_2_^−^.

The TC solution with a concentration of 50 mg L^−1^ and a 300 W xenon lamp (*λ* = 300–420 nm) were employed as the simulated contaminant and the light source, respectively, to evaluate the photocatalytic activity of BNNS, Bi_2_O_3_, and Bi_2_O_3_/BNNS composites for TC removal. As shown in Fig. 8a, a 60-minute dark adsorption step was first performed. The results confirm that 60 min is sufficient to reach adsorption equilibrium, ensuring that the subsequent concentration decrease under light irradiation is attributed solely to photocatalytic degradation. During the dark adsorption period, BNNS and Bi_2_O_3_/BNNS composites exhibited notably higher adsorption capacity for TC compared to Bi_2_O_3_. Furthermore, Fig. 8a illustrates that after 120 min of photocatalytic reaction, BNNS and Bi_2_O_3_ achieved removal rates of 58.43% and 38.62% for TC, respectively. The composites demonstrated removal rates above 80% under the same conditions, with Bi_2_O_3_/BNNS-3 exhibiting the highest efficiency at 94.65%. The results show relatively small error ranges (<5%), indicating that the experimental data possess good reproducibility and reliability.^38^ These results indicate that the superior performance of Bi_2_O_3_/BNNS-3 is not driven solely by BNNS adsorption, but rather originates from the synergistic effect between adsorption and photocatalysis. It is noteworthy that BNNS exhibits semiconductor characteristics, which suggest its potential for photocatalytic degradation. To assess the photocatalytic degradation rates of TC molecules, the first 80 minutes, during which the reaction rate is relatively high, was chosen as the evaluation time interval, employing the pseudo-first-order kinetic model for BNNS, Bi_2_O_3_, and Bi_2_O_3_/BNNS composites. Fig. 8b and c illustrate that the photocatalytic degradation processes of TC molecules for all materials adhere to the pseudo-first-order kinetic model. The calculated kinetic constants (*K*) for BNNS and Bi_2_O_3_ are 0.285 × 10^−2^ and 0.372 × 10^−2^ min^−1^, respectively. The kinetic constants of Bi_2_O_3_/BNNS composites are higher than BNNS and Bi_2_O_3_ due to the formation of a p–n junction. Specifically, the kinetic constant of Bi_2_O_3_/BNNS-3 is the highest at 2.177 × 10^−2^ min^−1^. The photocatalytic activity of BNNS is weak, therefore, with the further increase of BNNS content, the kinetic constants of the composites decrease, indicating a decline in photocatalytic activity.

To further explore the active species involved in the photocatalytic degradation of Bi_2_O_3_/BNNS composites, particularly Bi_2_O_3_/BNNS-3, a set of radical trapping experiments were conducted. PBQ, TEOA and IPA were utilized to capture the ·O_2_^−^, h^+^ and ·OH within the system. In Fig. 8d, it is evident that the introduction of TEOA had a negligible impact on the photocatalytic degradation of TC. Conversely, the presence of PBQ and IPA led to varying degrees of suppression in the TC degradation process, with suppression efficiencies of 54.21% and 39.76%, respectively. Therefore, the ·O_2_^−^ and ·OH contribute to the photocatalytic degradation of TC in the Bi_2_O_3_/BNNS composites system, with ·O_2_^−^ playing a crucial role. To further validate the involvement of these radicals, ESR spin-trapping experiments were carried out. As shown in Fig. 8e and f, characteristic signals of DMPO-·OH and DMPO-·O_2_^−^ were clearly observed under illumination, respectively, directly confirming the generation of hydroxyl radicals and superoxide radicals in the photocatalytic system. This outcome is consistent with the radical trapping results, further corroborating that ·OH and ·O_2_^−^ are the main reactive oxygen species responsible for TC degradation.

**Fig. 9:**
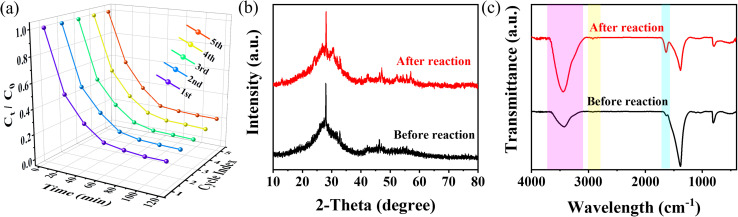
(a) Five cycles degradation curve of Bi_2_O_3_/BNNS-3 on TC solution; (b) XRD and (c) FT-IR patterns of Bi_2_O_3_/BNNS-3 before and after cycling.

Considering the cost of photocatalysts in practical applications is mainly dependent on the recycling ability of the material, this work conducted a further assessment of the photocatalytic degradation efficiency of TC using the Bi_2_O_3_/BNNS composites after four cycles of reuse. The detailed experimental procedure was as follows: after each photocatalytic reaction, the used Bi_2_O_3_/BNNS composite was collected by centrifugation, washed alternately with deionized water and absolute ethanol, dried, and then calcined at 150 °C for 2 h before being used in the next cycle. The findings from Fig. 9a illustrated that following four cycles, the photocatalytic degradation efficiency of Bi_2_O_3_/BNNS-3 remained at 93.64% of the initial value, demonstrating good reusability of the composite. Subsequently, the photocatalyst after four cycles was analyzed by XRD and FT-IR, comparing the data with the pre-cycling measurements. To evaluate the structural stability and assess the potential photocorrosion of Bi_2_O_3_, as depicted in Fig. 9b, there were insignificant alterations in the crystalline structure and phase composition of Bi_2_O_3_/BNNS-3 pre and post-reaction; no attenuation or emergence of new impurity peaks for the characteristic diffraction peaks of Bi_2_O_3_ was observed. A slight increase in crystallinity was observed after the reaction, likely attributed to the hydrolysis of certain unstable structures. This indicates that under the experimental conditions, no obvious photocorrosion of Bi_2_O_3_ occurred in the Bi_2_O_3_/BNNS composite, which can be attributed to the p–n heterojunction that facilitates electron transfer from BNNS to Bi_2_O_3_, effectively reducing hole accumulation on the Bi_2_O_3_ surface. Additionally, results from the FT-IR analysis (Fig. 9c) disclosed an augmentation in the intensity of O–H and C–H bonds in Bi_2_O_3_/BNNS-3 post-cycling, indicating the adsorption of a minor quantity of TC molecules or other degradation by-products on the catalyst surface. Nevertheless, the chemical structure and composition of the catalyst itself remained unaltered. Hence, the Bi_2_O_3_/BNNS composites exhibit remarkable structural stability and reusability.

**Fig. 10:**
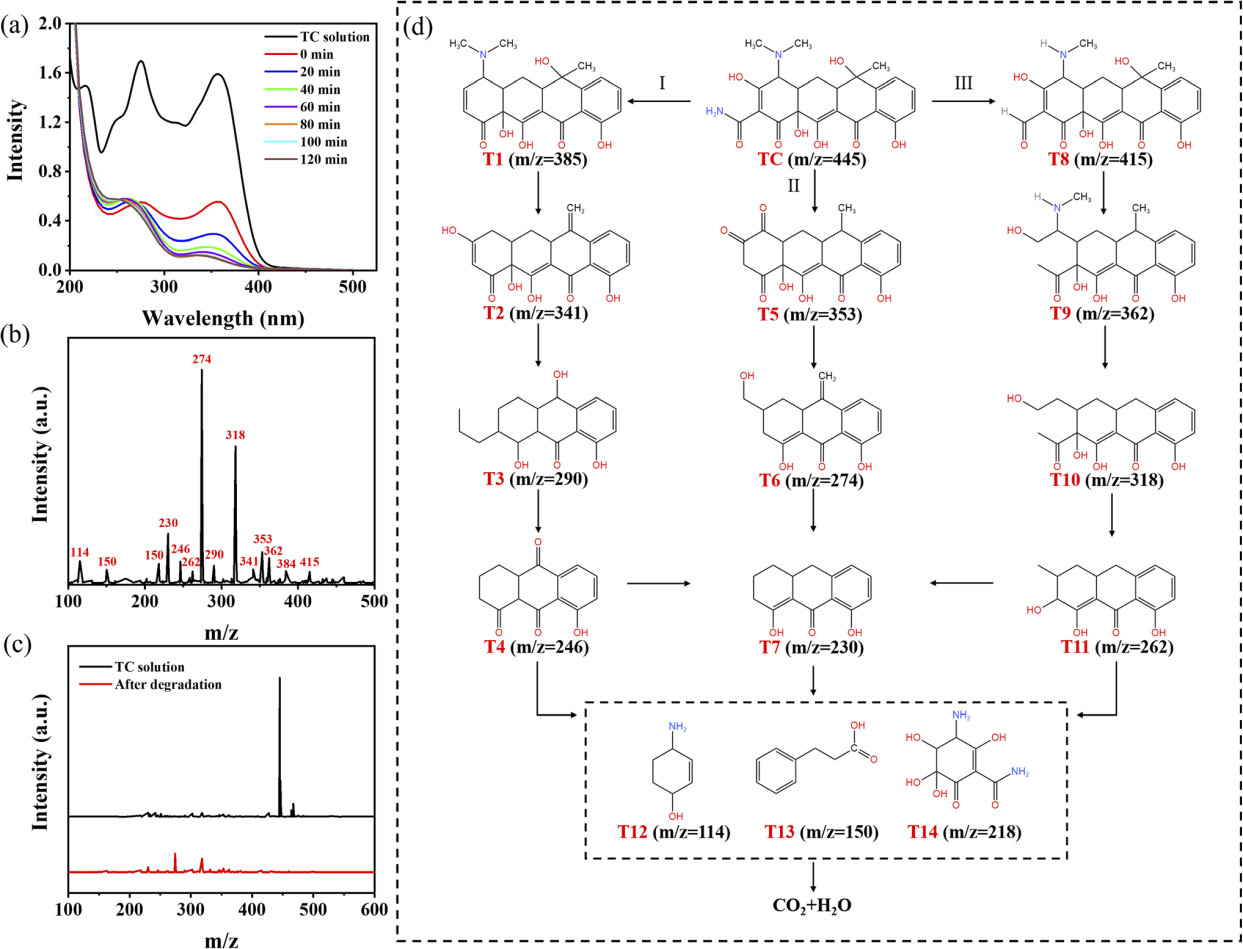
(a) UV-vis absorption spectra of TC solution during photocatalytic degradation; (b) liquid-phase mass spectra of the solution under light for 20 min, (c) liquid-phase mass spectra of the TC solution and the solution under light for 120 min and (d) presumed photocatalytic degradation process.

Using Bi_2_O_3_/BNNS-3 as a model, UV-vis spectra of the TC solution were analyzed during the degradation process. As illustrated in Fig. 10a, following 60 minutes of adsorption, a significant decrease in the UV-vis spectral intensity of the solution was observed. Subsequently, post-photocatalytic degradation, the UV-vis absorption spectrum of the solution exhibited substantial deviations from that of the TC solution, indicating the decomposition of TC molecules into other compounds. TC molecules, characterized by numerous double bonds, amino groups, aromatic rings, and phenolic structures, are prone to reactive free radical attacks.^39^ Employing liquid chromatography-mass spectrometry allows for the examination and analysis of intermediate components in the photocatalytic degradation process, facilitating a comprehensive investigation into the degradation mechanism of TC molecules. Fig. 10b illustrates the presence of a substantial number of intermediates in the liquid phase following 20 minutes of photocatalytic degradation. Fig. 10c presents the liquid-phase mass spectrometry images of the TC solution and the solution after 120 minutes of degradation. A comparative analysis reveals that the peak corresponding to the TC molecule (*m*/*z* = 445) has entirely vanished post-degradation, suggesting that the TC molecule has undergone oxidation and reduction to form various other substances through the action of active species. Finally, based on these experimental results, three potential degradation pathways for TC molecules are proposed in Fig. 10d. Pathway I initiates with the deamination of the TC molecule (*m*/*z* = 445), leading to the formation of T1 (*m*/*z* = 385) subsequent to ·OH radical attack. T1 then progresses into T2 (*m*/*z* = 341) and successively evolves into T3 (*m*/*z* = 290) and T4 (*m*/*z* = 246) through a sequence of ring-opening and molecular oxidation processes.^40^ In Pathway II, the interaction of the TC molecule with h^+^ and ·O_2_^−^ radicals triggers a cascade of dealkylation, ring-opening, and dealkylation reactions resulting in the production of T5 (*m*/*z* = 353), T6 (*m*/*z* = 274), and T7 (*m*/*z* = 230).^41^ In Pathway III, the relatively low N–C bond energy facilitates deamination and dealkylation of the TC molecule under the influence of ·O_2_^−^ and ·OH radicals, yielding T8 (*m*/*z* = 417). Following dehydration into an intermediate T9 (*m*/*z* = 362), it further undergoes dealkylation and ring-opening processes catalyzed by radicals to generate T10 (*m*/*z* = 318) and T11 (*m*/*z* = 262).^42^ As the photocatalytic oxidation progressed, the aforementioned intermediates were further transformed into lower molecular weight compounds, such as T12 (*m*/*z* = 114), T13 (*m*/*z* = 150), and T14 (*m*/*z* = 218),^43^ and were ultimately mineralized into water and carbon dioxide.

**Fig. 11:**
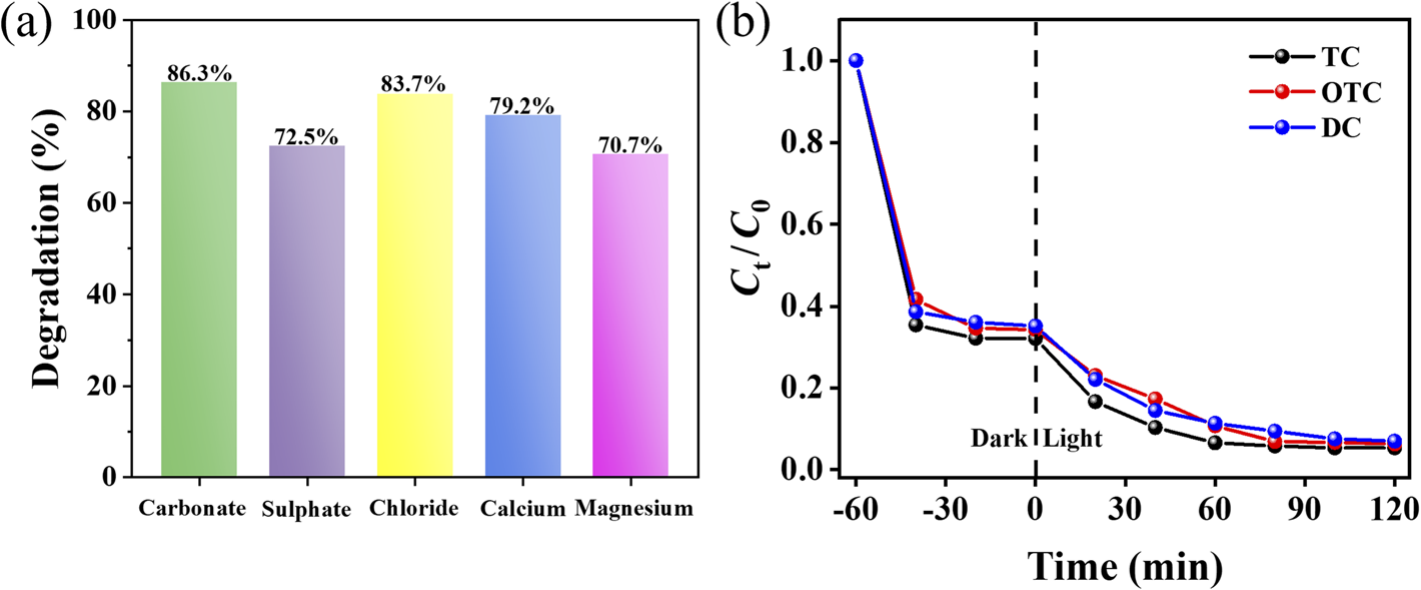
(a) TC degradation curves of Bi_2_O_3_/BNNS-3 in inorganic salt ion coexistence environment; (b) degradation curves of Bi_2_O_3_/BNNS-3 on TC, OTC and DC.

Industrial wastewater typically contains a diverse array of complex components, whereas the TC solution utilized in our experiments is characterized by high purity and the absence of extraneous substances. To better simulate real wastewater conditions and evaluate the practical applicability of the Bi_2_O_3_/BNNS-3, we conducted photocatalytic degradation experiments in the presence of typical groundwater ions at environmentally relevant concentrations: bicarbonate (100 mg L^−1^ as HCO_3_^−^), sulfate (50 mg L^−1^), chloride (50 mg L^−1^), calcium (50 mg L^−1^), and magnesium (20 mg L^−1^).^44–46^ The degradation efficiencies of tetracycline under different ion conditions are shown in Fig. 11a, which were as follows: bicarbonate (86.3%), chloride (83.7%), calcium (79.2%), sulfate (72.5%), and magnesium (70.7%). Bicarbonate and chloride exhibited relatively minor inhibitory effects on degradation efficiency, with removal rates remaining above 80%. In contrast, calcium, sulfate, and magnesium showed more pronounced suppression, which may be attributed to competitive adsorption between anions (particularly sulfate) and tetracycline molecules for the limited active sites on the catalyst surface, as well as the ion shielding effect induced by cations (calcium and magnesium). Despite the inhibitory effects of these coexisting ions, the Bi_2_O_3_/BNNS composite maintained a tetracycline removal rate of over 70% under all tested conditions, demonstrating its robust photocatalytic degradation capability and potential for real-world wastewater treatment applications. Furthermore, Bi_2_O_3_/BNNS-3 exhibits excellent photocatalytic degradation capabilities for both oxytetracycline (OTC) and doxycycline (DC) as shown in Fig. 11b. In conclusion, Bi_2_O_3_/BNNS composites possess significant photocatalytic degradation potential for practical applications.

**Fig. 12:**
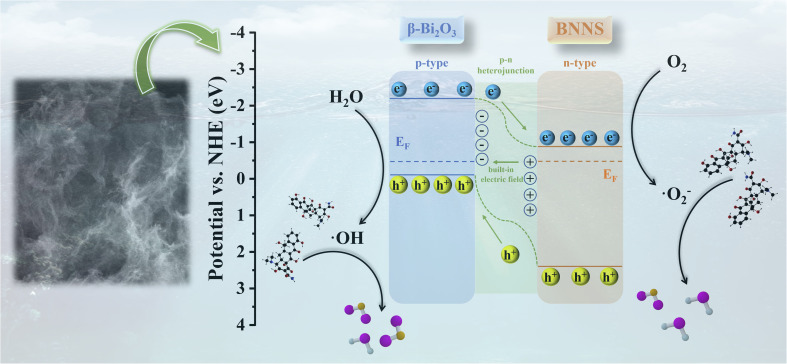
Photocatalytic degradation mechanism of TC by Bi_2_O_3_/BNNS composites.

Based on the previous discussion, the proposed mechanism for the photocatalytic degradation of tetracycline (TC) molecules using the Bi_2_O_3_/BNNS composites is illustrated in Fig. 12. In the composites, the p-type semiconductor Bi_2_O_3_ and the n-type semiconductor BNNS form a p–n junction. Due to the alignment of their Fermi levels, the band structures of both materials experience shifts, resulting in a more positive valence band and a more negative conduction band for the composite compared to each component. This alteration enhances the oxidation–reduction capabilities of the photogenerated electrons and holes. Under illumination, the photogenerated charge carriers in the two materials migrate due to the influence of the p–n junction: electrons generated in Bi_2_O_3_ transfer to the surface of BNNS, while holes produced in BNNS move to the surface of Bi_2_O_3_. This migration not only inhibits the recombination of photogenerated charge carriers but also establishes an internal built-in electric field within the catalyst, thereby facilitating an increased rate of charge carrier transfer. Moreover, the accumulation of photogenerated electrons and holes at the surfaces of BNNS and Bi_2_O_3_, respectively, results in the generation of superoxide radicals (·O_2_^−^) and hydroxyl radicals (·OH) on their surfaces. These free radicals subsequently engage in redox reactions with TC molecules, ultimately leading to their degradation into water, carbon dioxide, and other small molecular byproducts.

## 4. Conclusion

In this study, a solvent-thermal method was employed to fabricate high-performance Bi_2_O_3_/BNNS composites with synergistic adsorption–photocatalysis capabilities. The experimental results reveal that BNNS, serving as the growth substrate for Bi_2_O_3_ nanowires, effectively controls the growth and aggregation, resulting in enhanced specific surface area, pore volume, and robust adsorption capacity of the composites. Additionally, BNNS demonstrates n-type semiconductor properties, and the formation of a p–n junction and built-in electric field between Bi_2_O_3_ and BNNS promotes efficient separation of photogenerated electrons and holes within the catalyst, leading to superior photocatalytic activity of the composite material. Notably, both the adsorption capacity and photocatalytic degradation efficiency of the composite material outperform those of Bi_2_O_3_ and BNNS individually. Furthermore, the optimized concentration of Bi_2_O_3_/BNNS-3 at 50 mg L^−1^ achieves degradation rates exceeding 90% for TC, OTC, and DC solutions, with degradation performance unaffected by various impurity ions. Additional validation through free radical capture experiments, UV-vis spectroscopy, and liquid-phase mass spectrometry confirms that under simulated sunlight, the Bi_2_O_3_/BNNS composite material facilitates the oxidation of TC molecules into CO_2_, H_2_O, and other smaller compounds through the generation of ·OH and ·O_2_^−^. Overall, this research underscores the substantial potential of Bi_2_O_3_/BNNS composites in the photocatalytic degradation of water pollutants.

## Author contributions

Zhao Du: conceptualization, writing – original draft, writing – review & editing, investigation; Yanan Wu: visualization; Mengmeng Yu: formal analysis; Zishuang Cheng: project administration; Po Hu: validation verification; Zhonglu Guo: formal analysis; Chengchun Tang: project administration, resources; Guifeng Chen: supervision, project administration; Yi Fang: supervision, project administration.

## Conflicts of interest

There are no conflicts to declare.

## Data Availability

Data can be accessed upon reasonable request from the corresponding author at hebutdz@163.com.
